# Anti-aging Effects of Mangosteen Peel Extract and Its Phytochemical Compounds: Antioxidant Activity, Enzyme Inhibition and Molecular Docking Simulation

**DOI:** 10.21315/tlsr2020.31.3.9

**Published:** 2020-10-15

**Authors:** Wahyu Widowati, Chrismis Novalina Ginting, I Nyoman Ehrich Lister, Ermi Girsang, Annisa Amalia, Satrio Haryo Benowo Wibowo, Hanna Sari Widya Kusuma, Rizal

**Affiliations:** 1 Faculty of Medicine, Maranatha Christian University, Jl. Prof. drg. Surya Sumantri, No. 65, Bandung, West Java 40164, Indonesia; 2 University of Prima Indonesia, Jl. Sekip Jl. Sikambing No. Simpang, Sei Putih Tim. I, Kec. Medan Petisah, Kota Medan, Sumatera Utara 20111, Indonesia; 3 Biomolecular and Biomedical Research Centre, Aretha Medika Utama, Jl. Babakan Jeruk II No. 9, Sukagalih, Kec. Sukajadi, Kota Bandung, Jawa Barat 40163, Indonesia

**Keywords:** Antioxidant, Antiaging, Mangosteen, Phytochemical

## Abstract

Skin aging is a complex natural process characterised by gradual diminishment of structural integrity and physiological imbalance of the skin tissue. Since the oxidative stress is tightly corelated to the skin aging process, the usage of antioxidant may serve as favourable strategies for slowing down the skin aging process. Mangosteen is an important fruit commodity and its extract had been extensively studied and revealing various biological activities. Present study aimed to assess the antioxidant and antiaging activity of mangosteen peel extract (MPE) and its phytochemical compounds. MPE and its compounds were subjected to ferric reducing antioxidant power (FRAP), hydroperoxide (H_2_O_2_) scavenging, anti-collagenase, anti-elastase, anti-hyaluronidase and anti-tyrosinase assay. MPE has the highest FRAP 116.31 ± 0.60 μM Fe(II) μg^−1^ extract, IC_50_ of MPE on H_2_O_2_ scavenging activity was 54.61 μg mL^−1^. MPE also has the highest anti elastase activity at IC_50_ 7.40 μg mL^−1^. Alpha-mangostin showed potent anti-collagenase activity (IC_50_ 9.75 μg mL^−1^). While gamma-mangostin showed potent anti-hyaluronidase (IC_50_ 23.85 μg mL^−1^) and anti-tyrosinase (IC_50_ 50.35 μg mL^−1^). MPE and its compounds were evaluated *in vitro* for antioxidant and antiaging activities. Current findings may provide scientific evidence for possible usage of mangosteen extract and its compounds as antioxidant and antiaging agent.

**Highlight**

- MPE has flavonoid content around 17.66 μg QE mg-1 extract.
- Mangosteen peel extract showed potent antioxidant, anti-elastase and anti-collagenase activities, while gamma-mangostin showed potent antihyaluronidase and anti-tyrosinase.
- It was provide that mangosteen peel extract and its compounds could be used as antiaging agent.

## INTRODUCTION

Skin aging is a complex natural process characterised by a gradual diminishment of structural integrity and physiological imbalance of the skin tissue (Farage *et al*. 2013). The structural change caused by the degradation of extracellular matrix that underlies dermal tissue. There are various factors that affect the progression of skin aging which categorised into two groups, internal and external (Farage *et al*. 2008). Increased production of reactive oxygen species (ROS) is the main characteristic of cellular aging. Due to the nature of reactive oxygen species, radicals are highly reactive and can interact violently toward biological macromolecule. Skin fibroblast may undergo apoptosis when exposed directly toward radicals through activation of caspases pathway (Tanigawa *et al*. 2014). Not only affect cellular component of the skin, radical compound also holds an important role in the expression of protein related in degradation of extracellular matrix (Pittayapruek *et al*. 2016).

Since the oxidative stress is tightly corelated to the skin aging process, usage of antioxidant may serve as favourable strategies for slowing down the skin aging process (Masaki 2010). Previous study showed that the administration of plant extracts that are rich in antioxidant compounds can protect skin cell from cell death (Silverberg *et al*. 2011). Previously known plant extracts contain various phytochemical compounds that have various biological activities including antioxidants. Our previous studies showed strong antioxidant and antiaging activities *in vitro* in some plant extracts: White rice (*Oryza sativa*) (Widowati, Fauziah *et al*. 2016), jasmine flower (*Jasmimum sambac*) (Widowati *et al*. 2018), and rosella flower (*Hibiscus sabdari a*) (Widowati *et al*. 2017). This characteristic is the main reason underlies the plant utilisation as medicine. Mangosteen peel extract (MPE) had been extensively studied and revealing various biological activities (Widowati *et al*. 2014; Widowati, Fauziah *et al*. 2016, Gondokesumo *et al*. 2019; Widowati *et al*. 2020). These activities were likely attributed by the phytochemicals in mangosteen peel. Mangosteen peel was already characterised both physically and chemically, revealed various xanthones, including alpha-mangostin, gamma-mangostin, garcinone C, garcinone D, garcinone E, gartanin and smeathxanthone-A (Widowati *et al*. 2014; Gondokesumo *et al*. 2018; 2019). But to this date, antiaging benefit of mangosteen, especially its peel extract is very much unknown. To address this, we measured antioxidant properties and inhibitory activity against aging-related enzyme inhibition of MPE and its compounds. Present study describes the antioxidant and antiaging benefit of MPE.

## MATERIALS AND METHODS

### Preparation of Plant Extract

Plant material was collected from Mount Cisalak-Subang, West Java, Indonesia. Plant sample was identified by Drs. Djuandi, herbarium staff of Biology Department, School of Life Sciences and Technology, Bandung Institute of Technology, Bandung. Dried mangosteen peel was macerated in distilled ethanol (70%) overnight. The obtained filtrate was evaporated until became MPE, then diluted using Dimethyl sulfoxide (DMSO) [Sigma Aldrich, D4818, St. Louis, Missouri, United States] (Widowati, Fauziah *et al*. 2016; Widowati, Darsono *et al*. 2016; Widowati *et al*. 2017; 2018; Prahastuti *et al*. 2019).

### Total Flavonoid Content

Total flavonoid content in MPE was measured using aluminum chloride colorimetric assay (Prahastuti *et al*. 2019). Briefly 250 μL sample, 75 μL NaNO_2_ 5% [Sigma Aldrich, 67398, St. Louis, MO, US] and 150 μL AlCl_3_.H_2_O 10% [Sigma Aldrich, A0718, St. Louis, MO US] were mixed and incubated for 5 min. After incubated, 0.5 mL NaOH 1M [Sigma Aldrich, 221465, St. Louis, MO, US] was added. Absorbance was measured using spectrophotometer reader at 510 nm [Thermo, 51119200, Waltham, MA, US]. The results were reported as mg in quercetin equivalent (QE).

### Ferric Reducing Antioxidant Power (FRAP)

Antioxidant capacity of MPE and its compounds were determined based on reduction of Fe^3+^ ion (Widowati, Fauziah *et al*. 2016; Widowati *et al*. 2017; 2018). FRAP reagent were made by mixing 10 mL acetate buffer (300 mM, pH 3.6) [Sigma Aldrich S7899], 1 mL ferric chloride hexahydrate [Merck, 1.03943.0250, Kenilworth, NJ, US] 20 mM (dissolved in distilled water), and 1 mL of 2,4,6-Tris(2-pyridyl)-*s*-triazine (TPTZ) [Sigma Aldrich, 3682-35-7] (10 mM dissolved in HCl 40 mM [Sigma Aldrich, 320331]. In 96-well microplate, 7.5 μL sample was mixed with 142.5 μL FRAP reagent and incubated for 30 min in 37°C. The absorbance was measured at 593 nm using spectrophotometer [Thermo 51119200]. The standard curve was made using FeSO_4_ with concentration ranged from 0.019 to 95 μg mL^−1^. Results were reported as μM Fe (II)/μg extract (Widowati, Fauziah *et al*. 2016; Widowati *et al*. 2017; 2018).

### Peroxide Radical Scavenging Activity

Peroxide radical scavenging activity was measured using a method previously mentioned with slight modifications (Mukhopadhyay *et al*. 2016; Utami *et al*. 2017). The mixture was then transferred into 96-well plate and was incubated for 5 min at room temperature. Then 75 μL 1,10-phenanthroline was added to the mixture and incubated again for 10 min at room temperature. The absorbance was measured using a spectrophotometer at 510 nm using spectrophotometer [Thermo, 51119200, Waltham, MA, US].

### Elastase Inhibitory Activity

Elastase inhibitory activity was measured using a modified protocol from Sigma-Aldrich and Thring *et al*. (2009). Mixture of 10 μL sample with 5 μL porcine pancreas elastase [Sigma Aldrich, 45124] (0.5 mU mL^−1^, dissolved in cold distilled water), and 125 μL Tris buffer (100 mM, pH 8) was pre-incubated for 15 min at 25°C. 10 μL N-Sucanyl-Ala-Ala-Ala-p-Nitroanilide substrate [Sigma 54760, USA] (2 mg mL^−1^, dissolved in Tris buffer) was then added and incubated at 25°C for 15 min. Absorbance was measured at 410 nm using spectrophotometer [Thermo 51119200] (Tu & Tawata 2015; Widowati, Fauziah *et al*. 2016; Widowati *et al*. 2017; 2018).

### Collagenase Inhibitory Activity

Collagenase inhibitory activity was measured using a modified protocol from Sigma-Aldrich and Thring *et al*. (2009). Briefly 10 μL Collagenase from *Clostridium histolyticum* [Sigma Aldrich C8051] (0.01 U mL^−1^ in the cool aquadest), buffer Tricine 60 μL (50 mM, pH 7.5), sample 30 μL were mixed and incubated at 37°C for 20 min. After incubation N-[3-(2-Furyl)acryloyl]-leu-gly-Pro-Ala substrate substrate [Sigma Aldrich, F5135] (1mM in buffer *Tricine*) was added and incubated. Absorbance was measured at 335 nm using spectrophotometer [Thermo 51119200] (Thring *et al*. 2009; Widowati, Fauziah *et al*. 2016; Widowati *et al*. 2017; 2018).

### Tyrosinase Inhibitory Activity

Tyrosinase inhibitory activity was measured using a modified protocol from Tu and Tawata (2015). Briefly 20 μL sample, 140 μL potassium phosphate buffer (20 mM, pH 6.8), 20 μL mushroom tyrosinase (125 U mL^−1^ dissolved in potassium phosphate) were mixed and incubated at room temperature for 15 min. After incubated, 20 μL L-DOPA (1.5 mM) was added and the mixture was then incubated again at room temperature for 10 min. Absorbance was measured at 470 nm using spectrophotometer [Thermo 51119200] (Tu & Tawata 2015; Siregar *et al*. 2019).

### Hyaluronidase Inhibitory Activity

Hyaluronidase inhibitory activity was measured using a modified protocol from Tu and Tawata (2015). Briefly 25 μL sample, 3 μL bovine testes hyaluronidase type I-S [Sigma Aldrich H3506] was preincubated at 37°C for 10 min, 12 μL bufer fosfat (300 mM, pH 5.35) was added and incubated again at 37°C for 10 min. Afterward 10 μL substrate hyaluronic acid [Sigma Aldrich, H5542] was added and incubated at 37°C for 45 min. Hyaluronic acid decomposition was stopped by addition of 100 μL acidic albumin acid and incubated at room temperature for 10 min. Absorbance was measured at 600 nm using spectrophotometer [Thermo 51119200] (Tu & Tawata 2015; Widowati, Fauziah *et al*. 2016; Widowati *et al*. 2017; 2018).

### Molecular Docking Simulation

The possible binding mode of phytochemicals found in mangosteen peel towards protein related in aging process was modelled using molecular docking. The 3-dimensional structure of matrix metalloproteinase (MMP1), nuclear export protein (NEP), and prophenoloxidase (PPO3) were obtained from RCSB protein data bank, with PDBID 2Y9X, 5JMY, 966C, respectively. The data was then prepared by removing crystallographic water and removing any co-crystallised ligand. Molecular docking simulation was performed using AutoDock Vina (Vina) using default configuration (Trott & Olson 2010). Best docked conformation that ranked by vina scoring was used in the visual analysis using UCSF Chimera (Pettersen *et al*. 2004). Intermolecular interaction of protein-ligand complex was inferred using Pose View accessible through Protein Plus web server (https://proteins.plus/) (Stierand & Rarey 2010).

## RESULTS

### Flavonoid Content in Mangosteen Peel Extract (MPE)

Flavonoid content of MPE was measured. Present study found flavonoid content from MPE was around 17.66 μg QE mg^−1^ extract (Table 1). Previous study found mature mangosteen peel contains 4.08 g QE/100 g (Pothitirat *et al*. 2009). Thus, current finding showed higher concentration than previously found.

### Antioxidant Capacity Activity

Antioxidant work through reduction of radicals onto its neutral form. Thus, the reduction capacity of certain molecule attributed to its antioxidant activity (Baranowska *et al*. 2018). Antioxidant activity of MPE and its phytochemical was measured using colorimetric assay based on reducing capacity toward ferric ion. A compound with antioxidant activity will reduce Fe^3+^ into Fe^2+^. These ions will absorb different colour when complexed with TPTZ. The higher Fe^2+^ ion concentration indicating higher reducing capacity of a compound of interest has, thus describe its antioxidant activity. Present study found that MPE showed statistically very significant (*P* = 0.00) increase in reducing capacity as concentration rise compared to all other compounds in all concentrations. MPE was showed the highest antioxidant activity both in high (116.31 μg mL^−1^) and low (2.51 μg mL^−1^) concentration compared to its compounds alone (Table 2). Table 2 shows that MPE has a very good linearity which mean that this extract has very high antioxidant activity. Gamma-mangostin also showed good linearity in the data although the gradient is not quite high, that showed weak antioxidant activity. Garcinome C, garcinome D, and alpha-mangostin did not show good linearity and the gradient is very low (nearly 0) which can be robustly concluded that these compounds do not have antioxidant activity.

### Hydroperoxide Scavenging Activity

**Figure 1:**
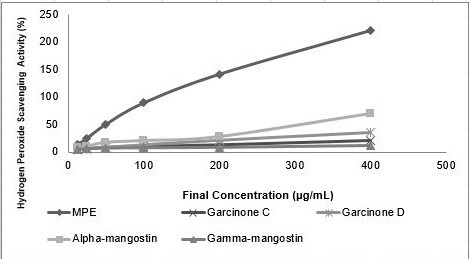
Hydro peroxide scavenging activity of MPE and its compounds. MPE, garcinone C, garcinone D, alpha-mangostin and gamma-mangostin were dissolved in DMSO to achieve final concentration of 12.5, 25, 50, 100, 200 and 400 (μg mL^−1^).

The hydroperoxide scavenging assay was carried out in order to measure antioxidant activity of the compound of interest, especially its activity against peroxide radicals. The assay was done using a colorimetric assay based on radical reduction and formation of coloured complexes (Mukhopadhyay *et al*. 2016; Utami *et al*. 2017; Jusri *et al*. 2019). Differ from previous method, the level scavenging activity of the extract and natural compound was measured based on their reduction activity toward peroxide radicals. Reduced radical was unable to oxidise ferric ions and Fe (II) will react directly with phenanthroline, forming a complex that has a strong orange colour which then quantified by a spectrophotometer (Mukhopadhyay *et al*. 2016; Utami *et al*. 2017; Jusri *et al*. 2019). In present study peroxide scavenging of MPE and its compounds were measured (Fig. 1). MPE showed the highest scavenging activity based on its IC_50_ value (54.61 μg mL^−1^) when compared to alpha-mangostin (120.52 μg mL^−1^), garcinone D (238.916 μg mL^−1^), garcinone C (437.64 μg mL^−1^) and gamma-mangostin (1051.45 μg mL^−1^) (Table 3).

### Elastase inhibitory Activity

**Figure 2:**
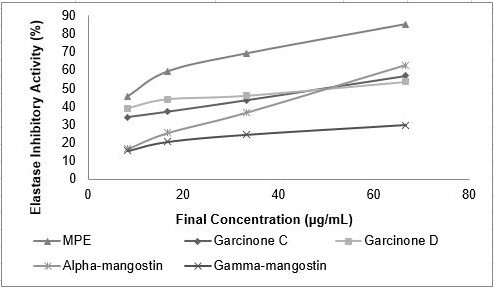
Elastase inhibitory activity of MPE and its compounds. MPE, garcinone C, garcinone D, alpha-mangostin and gamma-mangostin were dissolved in DMSO to achieve final concentration of 8.33, 16.67, 33.33 and 66.67 (μg mL^−1^).

In present study, anti-elastase of MPE and its compounds were measured (Fig. 2). MPE had the strongest inhibitory activity against elastase when compared with its known compound alone. Based on its IC_50_ value the inhibitory activity was increase as follows: gamma-mangostin < garcinone D < alpha-mangostin < garcinone C < MPE (Table 4).

### Collagenase Inhibitory Activity

**Figure 3:**
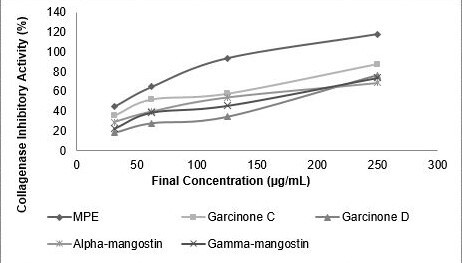
Collagenase inhibitory activity of MPE and its compounds. MPE, garcinone C, garcinone D, alpha-mangostin, gamma-mangostin were dissolved in DMSO to achieve final concentration of 31.25; 62.50; 125.00; 250.00 (μg mL^−1^).

The collagenase inhibition activity of MPE and its compounds were measured (Fig. 3). The strongest inhibitory activity achieved by alpha-mangostin (IC_50_ 9.75 μg mL^−1^) followed by MPE (IC_50_ 23.75 μg mL^−1^), gracinone C (IC_50_ 32.80 μg mL^−1^), alpha-mangostin (IC_50_ 55.41 μg mL^−1^), gamma-mangostin (IC_50_ 68.15 μg mL^−1^) (Table 5).

### Tyrosinase Inhibitory Activity

**Figure 4:**
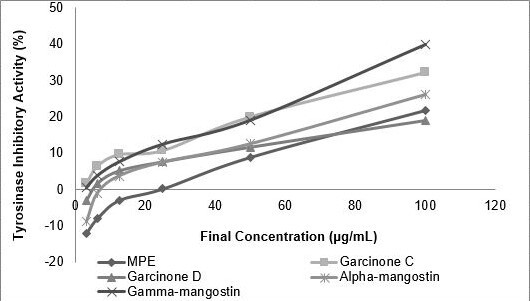
Tyrosinase inhibitory activity of MPE and its compounds. MPE, garcinone C, garcinone D, alpha-mangostin, gamma-mangostin were dissolved to achieve final concentration of 3.125, 6.25, 12.50, 25.00, 50.00 and 100.00 (μg mL^−1^).

Anti-tyrosinase activity of MPE and its compounds were measured in Fig. 4. Among sample tested, gamma-mangostin was found strongly inhibit tyrosinase activity (IC_50_ 50.35 μg mL^−1^) while MPE was the least (IC_50_ 181.08 μg mL^−1^). The inhibitory activity was increase as follows: MPE < garcinone D < alpha-mangostin < garcinone C < gamma-mangostin (Table 6).

### Hyaluronidase Inhibitory Activity

**Figure 5:**
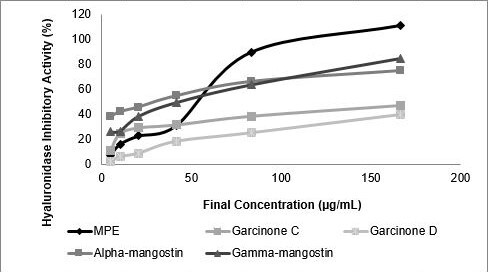
Hyaluronidase inhibitory activity of MPE and its compounds. MPE, garcinone C, garcinone D, alpha-mangostin, gamma-mangostin were dissolved in DMSO to achieve final concentration of 5.21, 10.42, 20.83, 41.67, 83.33 and 166.67 (μg mL^−1^).

The hyaluronidase inhibitotry activity of MPE and its compounds were measured (Fig. 5). Based on IC_50_ value alpha-mangostin had the strongest inhibitory activity against hyaluronidase when compared with another compound and MPE. The inhibitory activity was increase as follows: garcinone D < garcinone C < MPE < gamma-mangostin < alpha-mangostin (see Table 7).

### Molecular Docking

Molecular docking was performed to model the possible binding conformation of compounds found in mangosteen torwards MMP1, NEP and PPO3. Molecular docking was validated by redocking of bound ligand (N-hydroxy-2-[4-(4-phenoxy-benzenesulfonyl)-tetrahydro-pyran-4-yl]-acetamide, LBQ657, tropolone) to its respective protein. The RMSD of crystal conformation and docked conformation was less than the known cut off (> 2 Å, data not shown). Thus, the molecular docking method used was reasonable and continued with phytochemicals used in present study.

All compound was successfully docked to the receptor. The binding affinity was retrieved and compared to each other (Table 8). Alpha-mangostin has the highest binding affinity towards MMP1 (−8.9 kcal mol^−1^). Gamma-mangostin has the highest binding affinity towards NEP (−7.8 kcal mol^−1^). While gamma-mangostin has the bind strongly towards PPO3 (−6.8 kcal mol^−1^). None of the compounds exceeded binding affinity of bound ligand, except for gamma-mangostin towards PPO3.

**Figure 6:**
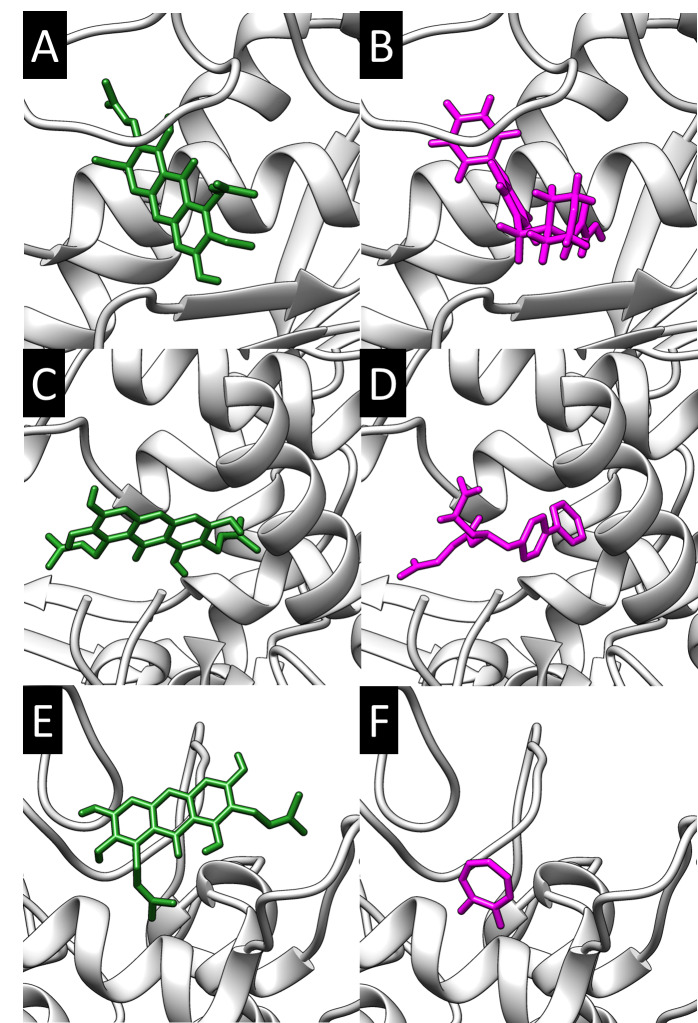
Molecular docking of phytochemical compounds against MMP1, NEP, and PPO3. (A) alpha-mangostin and (B) N-hydroxy-2-[4-(4-phenoxy-benzenesulfonyl)-tetrahydro-pyran-4-yl]-acetamide towards MMP1. (C) gamma-mangostin and (D) LBQ657 towards NEP. (E) gamma-mangostin and (F) tropolone towards PPO3. The protein showed as surface representation. The ligand showed as stick representation with only polar hydrogen showed.

The binding conformation with highest binding affinity was then visualised in Fig. 6. Visualisation analysis showed that all the potential compound was occupied the active site of the receptor, like the bound ligand.

## DISCUSSION

The biological activities of plant extract often attributed to its phytochemical content. Plant especially its peel often characterised with enormous amount phytochemical compound. Mangosteen (*Garciana mangostana* L.) (Clusiaceae) peel previously known to be rich in polyphenolic compound belong to xanthone group. There were 40 xanthones present in the pericarp of the mangosteen fruit, the most abundant xanthones found are alpha-mangostin, beta-mangostin and gamma-mangostin (Chen *et al*. 2008; Pedraza-Chaverrí *et al*. 2009; Zarena & Sankar 2009). Mangostins (alpha, beta and gamma) are the most frequently studied. They have a unique chemical structure with a tricyclic aromatic system carrying isoprene, hydroxyl and methoxyl groups (Obolskiy *et al*. 2010). Phytochemical found in mangosteen peel includes garcinone, mangostin, isomangostin and garcimangosone (Obolskiy *et al*. 2010). These compounds have been reported to possess anti-oxidant, anti-proliferative, pro-apoptotic, anti-inflammatory and anti-carcinogenic activities in *in vitro* and *in vivo* studies (Gutierrez-Orozco & Failla 2013; Widowati *et al*. 2017; 2020). Present study found MPE has higher flavonoid concentration than previously described. The phytochemical content of plant extract may different because of various factors, including seasonal or nutritional factors.

Aging is a natural process that affects various organs of the body and often characterised the build-up of ROS in cells (Davalli *et al*. 2016). ROS in normal circumstance holds an important role in various biological process, such as immune response, but through various stimulus or age, the homeostasis of radical was impaired. Increased cytoplasmic ROS is able to induce the synthesis of related to the degradation of extracellular matrix causing tissue structural diminishment that manifested as formation of wrinkles and sagging elasticity (Farage *et al*. 2008). Antioxidant phytochemical compound helps decrease ROS induced skin damages (Tanigawa *et al*. 2014). Not only relives the oxidative stress, administration phytochemical compound followed by the decreased collagen degrading enzyme activity (Tsai *et al*. 2014). Present study found that all compound of MPE have strong antioxidant potency based on its measured reductive capacity. Thus, MPE may alleviate skin cells damage caused by oxidative stress. MPE may also indirectly attenuates the activity of enzyme-related in the degradation of extracellular matrix.

Previous study shows that increasing ROS generation induces expression of various enzyme, including elastase. Human skin elastase is a serine protease responsible elastin degradation process. Chronic irradiation of UVB increased elastase activity significantly, causing the disintegration of elastin fibre in the dermal matrix and further manifested the loss of the skin elasticity. Previous study found *Zingiber officinale* (L.), extract inhibit the human skin fibroblast elastase activity (Imokawa & Ishida 2015). Another study measured the anti-elastase activity of 21 plant extracts (Thring *et al*. 2009). Present study shows MPE has a potent anti-elastase activity even when compared with its phytochemical alone. Present finding may serve as a mechanistic explanation of previous study, where oral administration of MPE was found increased skin elasticity (Ohno *et al*. 2015).

The tensile strength of the skin was provided by the collagen fibre present in dermal matrix. The synthesis of matrix metalloproteinase (MMPs), an enzyme involved in collagen hydrolysis, plays important role in the progression of skin aging (Pittayapruek *et al*. 2016). Since elevated MMPs synthesis was found to be correlated to the formation of skin changes, especially wrinkles. Several plant extracts can inhibit MMPs activity, especially white tea extract which contains the highest phenolic (Thring *et al*. 2009). In present study, alpha-mangostin has the highest inhibition activity towards MMPs. Thus, showing the potency of mangosteen phytochemicals for hampering the progression of wrinkles formation.

Hyaluronic acid (HA) is an example of glycosaminoglycans (GAGs) that plays an important role in maintaining skin the moisture of the skin (Stern & Maibach 2008). HA is a strong hydrophilic compound that can absorb 1000x of water volume it has (Baumann 2007). The degradation of HA related to the loss of skin moisture, as usually found on aging skin. In addition, GAGs are also served as peripheral proteins for other structural proteins such as collagen and elastin (Baumann 2007). Thus, the loss was also found to contribute to diminished dermal integrity. The result show gamma-mangostin inhibit hyaluronidase. This finding may explain how consumption of mangosteen peel extract helps to maintain skin moisture as can be found in previous study (Ohno *et al*. 2015).

Besides the structural changes, aging stimulus was able to induce darkening of the skin or often called hyperpigmentation (Ebanks *et al*. 2009). Skin pigmentation caused by the production of melanin pigment on the dermal melanocyte. This process is regulated by the rate-limiting enzyme tyrosinase, which serves as an important target for preventing pigmentation. Natural compound was already used commercially as anti-browning agent and skin whitening. Kojic acid works through the inhibition of tyrosinases functions since its share similar structure with its substrate (Chen *et al*. 1991). Current study found gamma-mangostin strongly inhibit tyrosinase activity. Thus gamma-mangostin may serve as potential skin whitening agent in further study.

To further investigate possible interaction between phytochemicals found in MPE towards enzyme related in aging process previously described, molecular docking was performed. Molecular docking of phytochemicals found in mangosteen peel predicted alpha-mangostin was a potential binder towards MMP1 while gamma-mangostin towards NEP and PPO3. Visual analysis showed that docked compound reside the active site cavity of the respective protein. Thus, present study proposed the possible binding conformation of alpha-mangostin towards MMP1 and gamma-mangostin towards NEP and PPO3.

Our results show that pure active compound of mangosteen such as alpha-mangostin, gamma-mangostin, garcinone C, and garcinone D had lower activity than MPE, even though they are the active compounds of MPE. These possibly caused by the reactivity of polyphenolic compounds that readily reacted with components of cell culture media to interact with H_2_O_2_, quinones, and semiquinones, and to up regulate the antioxidant defense(Gutierrez-Orozco & Faila 2013). Study done by Gutierrez-Orozco *et al*. (2013) showed that alpha-mangostin readily degraded in differenty types of serum-free media (i.e., RPMI, DMEM, MEM and McCoy’s 5A) when added in a dimethyl sulfoxide (DMSO) stock solution (Gutierrez-Orozco *et al*. 2013).

## CONCLUSION

MPE and its compounds were evaluated *in vitro f*or antioxidant and antiaging activities. Mangosteen peel extract showed potent antioxidant, anti-elastase and anti-collagenase activities, while gamma-mangostin showed potent anti-hyaluronidase and anti-tyrosinase. Current findings may provide scientific evidence for possible usage of mangosteen peel extract and its compounds as antiaging agent.

## Figures and Tables

**Table 1:** Flavonoid content in MPE.

	Total flavonoid (μg mg^−1^ extract)
Mangosteen peel extract	17.66 ± 0.19

**Table 2:** Antioxidant capacity of MPE and its compounds.

FRAP-reducing Activity (μM Fe(II) μg^−1^ sample)					

Concentration (μg mL^−1^)	MPE	Garcinone C	Garcinone D	Gamma-mangostin	Alpha-mangostin
25.00	116.31 ± 0.60^f^	2.51 ± 0.04^e^	2.20 ± 0.25^e^	3.69 ± 0.73^d^	1.79 ± 0.13^c^
12.50	56.75 ± 4.06^e^	2.30 ± 0.14^e^	1.81 ± 0.15^d^	2.86 ± 0.55^cd^	1.65 ± 0.11^c^
6.25	31.16 ± 0.42^d^	1.89 ± 0.12^d^	1.77 ± 0.04^cd^	2.28 ± 0.33^bc^	1.57 ± 0.29^c^
3.13	14.87 ± 0.35^c^	1.33 ± 0.12^b^	1.44 ± 0.08^bc^	1.58 ± 0.10^ab^	1.17 ± 0.04^b^
1.56	8.68 ± 1.03^b^	1.12 ± 0.08^ab^	1.28 ± 0.01^a^	1.19 ± 0.12^a^	1.04 ± 0.02^b^
0.78	4.48 ± 0.75^ab^	0.96 ± 0.08^b^	1.17 ± 0.08^a^	1.03 ± 0.13^a^	0.88 ± 0.06^ab^
0.39	2.51 ± 0.25^a^	0.61 ± 0.06^a^	0.77 ± 0.12^a^	0.98 ± 0.03^a^	0.57 ± 0.05^a^

* The data presented as mean ± SD of triplicate experiment. (MPE, garcinone-C, garcinone-D, gamma-mangostin, alpha-mangostin) are not significantly difference among concentrations of samples at *p* < 0.05 (Tukey’s range test).

**Table 3:** Hydro peroxide scavenging activity of MPE and its compounds.

Sample	Linear equation	*R^2^*	IC_50_ (μM)	IC_50_ (μg mL^−1^)
MPE	y = 0.5231x + 21.432	0.97	-	54.61
Garcinone C	y = 0.0424x + 5.1783	0.97	1057.12	437.64
Garcinone D	y = 0.0822x + 4.1658	0.99	557.59	238.916
Gamma-mangostin	y = 0.0424x + 5.1783	0.97	2652.25	1051.45
Alpha-mangostin	y = 0.15x + 5.9557	0.96	293.63	120.52

**Table 4:** Elastase inhibitory activity of MPE and its compounds.

Sample	Linear equation	*R^2^*	IC_50_ (μM)	IC_50_ (μg mL^−1^)
MPE	y = 0.626x + 45.36	0.94	-	7.40
Garcinone C	y = 0.395x + 30.59	0.99	49.11	20.33
Garcinone D	y = 0.230x + 38.51	0.96	49.79	21.33
Gamma-mangostin	y = 0.218x + 15.77	0.93	156.35	61.98
Alpha-mangostin	y = 0.770x + 11.44	0.99	50.01	20.53

**Table 5:** Collagenase inhibitory activity of MPE and its compounds.

Sample	Linear equation	*R^2^*	IC_50_ (μM)	IC_50_ (μg mL^−1^)
MPE	y = 0.32x + 42.38	0.93	-	23.75
Garcinone C	y = 0.22x + 32.57	0.96	79.22	32.80
Garcinone D	y = 0.259x + 8.66	0.97	159.04	68.15
Gamma-mangostin	y = 0.216x + 19.69	0.96	139.76	55.41
Alpha-mangostin	y = 0.173x + 27.42	0.95	23.76	9.75

**Table 6:** Tyrosinase inhibitory activity of MPE and its compounds.

Sample	Regression Linear	*R^2^*	IC_50_(μM)	IC_50_(μg mL^−1^)
MPE	y = 0.33x + 9.520	0.96	-	181.08
Garcinone C	y = 0.23x + 3.81	0.97	158.61	65.66
Garcinone D	y = 0.196x + 0.51	0.90	251.73	107.86
Gamma-mangostin	y = 0.38x + 1.30	0.99	127.00	50.35
Alpha-mangostin	y = 0.31x + 3.51	0.91	172.79	70.92

**Table 7:** Hyaluronidase inhibitory activity of MPE and its compounds.

Sample	Linear equation	*R^2^*	IC_50_(μM)	IC_50_(μg mL^−1^)
MPE	y = 0.6679x + 9.5863	0.92	-	60.51
Garcinone C	y = 0.1384x + 25.052	0.96	180.26	74.62
Garcinone D	y = 0.2238x + 4.2644	0.96	204.36	87.56
Gamma-mangostin	y = 0.3639x + 28.105	0.95	60.17	23.85
Alpha-mangostin	y = 0.2222x + 41.233	0.91	39.46	16.19

**Table 8:** Binding affinity of docked mangosteen peel compounds.

Compound	Binding affinity (kcal mol^−1^)		

	MMP1	NEP	PPO3
Garcinone C	−8.1	−7.6	−6.4
Garcinone D	−8.2	−7.3	−6.1
Gamma-mangostin	−8.5	−7.8	−6.8
Alpha-mangostin	−8.9	−7.4	−6.5
Bound ligand	−9.9	−9.0	−5.8
